# Modeling the Epidemiological Trend and Behavior of COVID-19 in Italy

**DOI:** 10.7759/cureus.9884

**Published:** 2020-08-20

**Authors:** Alessandro Rovetta, Akshaya S Bhagavathula, Lucia Castaldo

**Affiliations:** 1 Mathematical, Statistical and Epidemiological Models, Research and Disclosure Division, Mensana Srls, Brescia, ITA; 2 Mathematical, Statistical and Epidemiological Models, Technological and Scientific Research, Redeev Srl, Naples, ITA; 3 Public Health, Institute of Public Health, College of Medicine and Health Sciences, United Arab Emirates University, Al Ain, ARE; 4 Mathematics, Technological and Scientific Research, Redeev Srl, Naples, ITA; 5 Mathematics, Research and Disclosure Division, Mensana Srls, Brescia, ITA

**Keywords:** covid-19, pandemic, contamination, novel coronavirus, sars-cov-2, europe, italy

## Abstract

As of May 14, 2020, Italy has been one of the red hotspots for the COVID-19 pandemic. In particular, the regions of Emilia Romagna, Piedmont, and especially Lombardy were the most affected and had to face very serious health emergencies, which brought them to the brink of collapse. Since the virus has demonstrated local properties, i.e., greater severity and contagiousness in specific regions, the aim of this study is to model the complex behavior of severe acute respiratory syndrome coronavirus 2 (SARS-CoV-2) in Italy. In particular, we further investigated the results of other articles on the correlation with particulate matter pollution 10 (PM 10) and 2.5 (PM 2.5) by extending the research at the intra-regional level, as well as calculated a more plausible number of those infected compared to those officially declared by Civil Protection. Through a computational simulation of the Susceptible-Exposed-Infectious-Recovered (S.E.I.R.) model, we also estimated the most representative basic reproduction number $R0$ for these three regions from February 22 to March 14, 2020. In doing so, we have been able to evaluate the consistency of the first containment measures until the end of April, as well as identify possible SARS-CoV-2 local behavior mutations and specificities.

## Introduction

The current surge of COVID-19 pandemic is devastating globally, with over 4,200,000 cases and more than 290,000 deaths reported [1]. In Europe, COVID-19 cases have started to dramatically increase from the first week of March 2020. Of these, Italy was grappling with the worst outbreak, with over 35,713 confirmed cases, and around 3000 confirmed deaths by March 18, 2020 [2]. This exponential increase in COVID-19 positive cases in Italy raised turmoil, and the government decreed a lockdown for the entire country [3].

This is the first study that examines the behavior of severe acute respiratory syndrome coronavirus 2 (SARS-CoV-2) at the provincial level in the most affected regions. Considering that researches on a national scale are subject to high variances, as they use regional data, i.e., consider the regions as uniform epidemic nuclei, such a survey is fundamental to understand, which is the most appropriate investigation scale between the national, regional, and provincial ones. Making use of the epidemic parameters provided by the World Health Organization (WHO), we utilized the Susceptible-Exposed-Infectious-Recovered (S.E.I.R.) mathematical model to predict the trend of infections during the first half of March 2020, when the effects of the lockdown were not yet measurable. This allowed us to signal the presence of SARS-CoV-2 local characteristics changes due to possible evolutionary genetic mutations, correlations with pollution like Particulate Matter 10 (PM 10) and Particulate Matter 2.5 (PM 2.5), mismanagement of the crisis by national government agencies, and non-compliance with the lockdown rules by citizens or other unknown factors. To do this, we compared the general trend foreseen by the S.E.I.R. with the estimated, highlighting the discrepancies between the individual Italian regions.

## Materials and methods

To carry out this study, the most recent data found in the scientific literature relating to the total and active cases, deaths, recoveries, and all epidemic parameters of COVID-19 have been used. We focused especially on Lombardy since it was by far the most afflicted region in Italy. Considering the novel coronavirus incubation period is around three to six days, with a range from a minimum of two days to a maximum of 14, we first examined the population of COVID-19 cases reported between February 22 and March 14, 2020 [4]. Following this, we analyzed the Pearson linear correlation between the number of COVID-19 total cases and the concentrations of PM 10 and PM 2.5. All daily PM data were collected from the Regional Agency for Environmental Protection (ARPA) regional websites in the interval of January 1 - May 14, 2020. Several monitoring stations were used, and all results were provided with a Gaussian 95% confidence interval. All data have been organized into two time periods such as "pre-lockdown," from January 1 until February 29, 2020, and “post-lockdown,” from March 1 until May 14, 2020; this helped us estimate the link between the virus spread and the particulate matter (as emissions dropped dramatically during the lockdown). Other types of correlation with population density and number of inhabitants were also investigated.

S.E.I.R. modeling

Assuming as true the probable "non-relapse patients" hypothesis, we applied the S.E.I.R. model to predict the novel coronavirus evolution in Italy, as it is suitable for describing the spread of a virus in populations where no restrictions have been applied [5]. Thanks to the comparison between the S.E.I.R. values and the theoretical estimates (TE) in the short period, it is likely to highlight essential behavior mutations and/or the effectiveness of containment strategies. We used S.E.I.R. differential equations and non-linear methods to resolve the gaps analytically [6]. We examined Lombardy, Emilia Romagna, and Piedmont separately because of the huge discrepancy of COVID-19 cases between these and other Italian regions. An iterative algorithm was developed using the C++ programming language to find a solution through a finite discretization method (Appendix 1). Given the low deaths-population ratio, the total population number has been considered constant.

Software iterative algorithm

By entering the initial values for the incubation time $1/\sigma$, the recovery time $1/\gamma$, the basic reproduction number $R0$, the number of infected $I0$, and the number of recovered $R0$ on February 22, 2020, the software prints the S.E.I.R. predictions day by day. The best epidemic parameters were estimated through continuous iteration until the "closest values to the real ones" were reached until March 12, 2020. The number of initial incubates was calculated with the formula $E0 = R0 \cdot I0$. We report below the system of equations and their discretization through the finite increment $\delta t$:

$\begin{bmatrix} S'(t) = \beta \cdot I \cdot S/N\\ E'(t) = \beta \cdot I \cdot S/N - \sigma \cdot E\\ I'(t) = \sigma \cdot E - \gamma \cdot I\\ R'(t) = \gamma \cdot I\\ N'(t) = 0 \end{bmatrix}$

$\begin{bmatrix} S_{i+1} = \beta \cdot I_i \cdot S_i/N_i \cdot \delta t + S_i\\ E_{i+1} = (\beta \cdot I_i \cdot S_i/N_i - \sigma \cdot E_i) \cdot \delta t + E_i\\ I_{i+1} = (\sigma \cdot E_i - \gamma \cdot I_i) \cdot \delta t + I_i\\ R_{i+1} = \gamma \cdot I_i \cdot \delta t + R_i\\ N_i = S_i + E_i + I_i + R_i \end{bmatrix}$

with $1/\gamma$ incubation time, $1/\sigma$ recovery time, $R0$ basic reproduction number, $S$ number of susceptible people, $E$ number of active exposed people (people in incubation), $I$ number of active infected people, $R$ number of recovered people (no longer infectable).

$R0$ statistical analysis

The compatibility between S.E.I.R. predictions $x_i$ and TE values $X_i$ was investigated within a closed ball of radius five days center on March 7 (from March 2 to March 12, 2020). We searched for the best infection mortality rate $m$ and basic reproduction number $R0$ through the minimization of two estimators: the first, called $\Delta$, was the algebraic mean value of the absolute percentage differences $\delta_i$ between the best value $X_i$ for the $x_i$ S.E.I.R. value Gaussian distribution $G(X_i, \sigma_i)$ and the $x_i$ value itself, according to the formula $\delta_i = |X_i - x_i| / x_i$. This allows us to assess the quality of S.E.I.R. modeling. The second, called $\varepsilon$, was the algebraic mean value of the ratios $\beta_i$ between the fixed standard deviation $\sigma_i = \Big[(n-1)^{-1}\cdot\sum _{i=1}^n (X_i - x_i)^2\Big]$ and the corresponding $x_i$, according to the formula $\beta_i = \sigma_i / x_i$. This allows us to calculate the $x_i$ relative errors. The lower the $\Delta$, the more representative $R0$ is of the TE evolution; the lower the $\varepsilon$, the more accurate is the TE. The iteration was carried out until $\varepsilon$ and $\Delta$ minimums were reached. The finite increments chosen for $m$ and $R0$ were $\delta m = 0.2$ and $\delta R0 = 0.1$, respectively. Every combination with m in $[0.7, 1.5]$ and $R0 \in [1,7]$ was tried. The chosen significance limit for $\varepsilon$ and $\Delta$ was $0.1$ (i.e. $\Delta$ and $\varepsilon$ must be lower than or equal $0.1$ for a result to be acceptable); the iteration ended when $\Delta$ and $\varepsilon &lt; 0.05$. The best $R0$ confidence intervals were calculated considering the Gaussian distribution $G(R0, 2\delta R0)$. We reported a range interval “CRI” for all compatibles $R0$ we found. All the analysis was carried out through C ++ software and Microsoft Excel (Microsoft Corporation, Redmond, Washington). Since $m$ is subject to a very wide margin of error; when more than one $(m, R0)$ compatible couple was computed, we utilized the weighted average $m_{best} = \Big(\sum _{i=1}^n m_i w_i\Big) \cdot \Big(\sum _{i=1}^n w_i\Big)^{-1}$.

COVID-19 real cases estimate

In order to calculate the real number of COVID-19 total cases in Lombardy, we used an estimation method we called "Theoretical Estimate" (TE). Thanks to the results of another study, in which the number of regional and national deaths until May 5, 2020, was compared with those of the previous five years, it was possible to calculate the number of theoretical cases by adding the number of COVID-19 missing cases until the expected infection mortality was achieved. To do this, we considered a mortality rate free to vary in the range (0.6, 1.6) [7-9]. The “COVID-19 total deaths number” was shifted seven days backward due to the time between contracting the disease and demise. We also calculated the difference between confirmed and estimated COVID-19 trough the ratio α = estimated cases / confirmed cases.

PM 10 data analysis

First, we collected PM 10 daily averages data on the most affected cities of the three main regions involved in the COVID-19 epidemic such as Lombardy, Piedmont, and Emilia Romagna; then, we did the same for some regions where the novel coronavirus spree was not so pressing like Lazio and Campania. Finally, we built the symmetric correlation matrix $\rho_{ij}$:

$\begin{bmatrix} 1 & \rho_{12} & \rho_{13} & \rho_{14} \\ & 1 & \rho_{23} & \rho_{24} \\ & & 1 & \rho_{34} \\ & & & 1 \end{bmatrix}$

with $\rho_{12}$ PM 10 - COVID-19 confirmed cases Pearson correlation index, $\rho_{13}$ PM 10 - population density Pearson correlation index, $\rho_{14}$ PM 10 - total population Pearson correlation index, $\rho_{23}$ COVID-19 cases - population density Pearson correlation index, $\rho_{24}$ PM 10 - COVID-19 Pearson correlation index, $\rho_{34}$ population density - total population Pearson correlation index. 

However, since the first outbreak occurred in northern Italy, it is plausible to think the infection did not have the same contagion power in the southern regions; thus, we recalculated the matrix $\rho$ only for the three most infected regions to check whether there was a local correlation. Finally, in order to make the investigation even more precise and specific, we repeated the operation once again in all Lombardy provinces. All the values $\rho_{ij}$ have been reported with their relative p-values, according to the form $\rho_{ij}$ (ij-th p-value). All the PM 10 average daily values were reported with Gaussian 95% confidence intervals $(AV-2\cdot\sigma/\sqrt{N}, AV+2\cdot \sigma/\sqrt{N})$.

PM 2.5 data analysis

While other studies have been conducted on the PM 2.5 - novel coronavirus correlation at the national level, we have focused exclusively on the Lombardy region, analyzing the data of all the monitoring units of all the provinces through the previously defined $\rho$ correlation matrix [10].

## Results

Epidemic forecast

**Figure 1:**
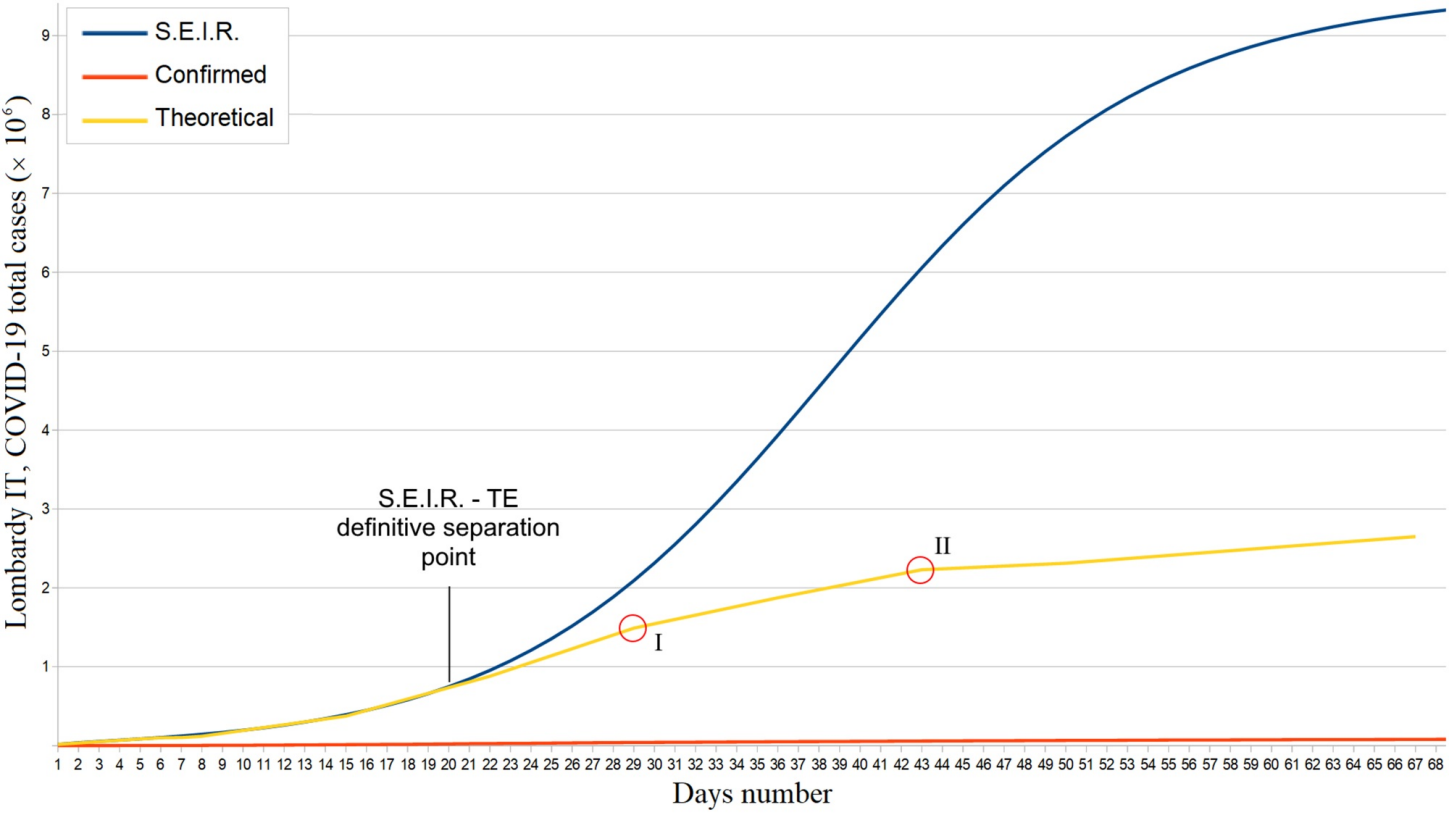
Lombardy confirmed, theoretical, and S.E.I.R. simulation total COVID-19 cases trends from February 22 to May 1, 2020 S.E.I.R. = Susceptible - Exposed - Infectious - Recovered; TE = Theoretical Estimate

For each infection mortality rate m, a compatible value of $R0$ was found; therefore, we utilized the weighted average $m = 0.011\; (95\% CI : 0.006 - 0.016)$. For the Lombardy region in the period February 22 - March 12, 2020, we estimated a basic reproduction number $R0 = 3.91\;(95\% CI: 3.87 - 3.94, CRI: 3.82 - 3.91)$, with $\Delta = 0.02\;(95\% CI: 0.01 - 0.03)$ and $\varepsilon = 0.04\;(95\% CI: 0.03 - 0.05)$. The calculated number of real infections exceeds that of confirmed infections by a factor $\alpha \sim 34$ until May 1, 2020 $(\alpha = 34.1, 95\% CI: 33.0 - 35.3)$. The separation point between the S.E.I.R. and TE trends is positioned in a neighborhood of March 12, 2020; ergo, that is the period when the lockdown began to take effect (Figure 1). The two corner points I and II in Figure 1 indicate further decreases in $R0$.

**Figure 2:**
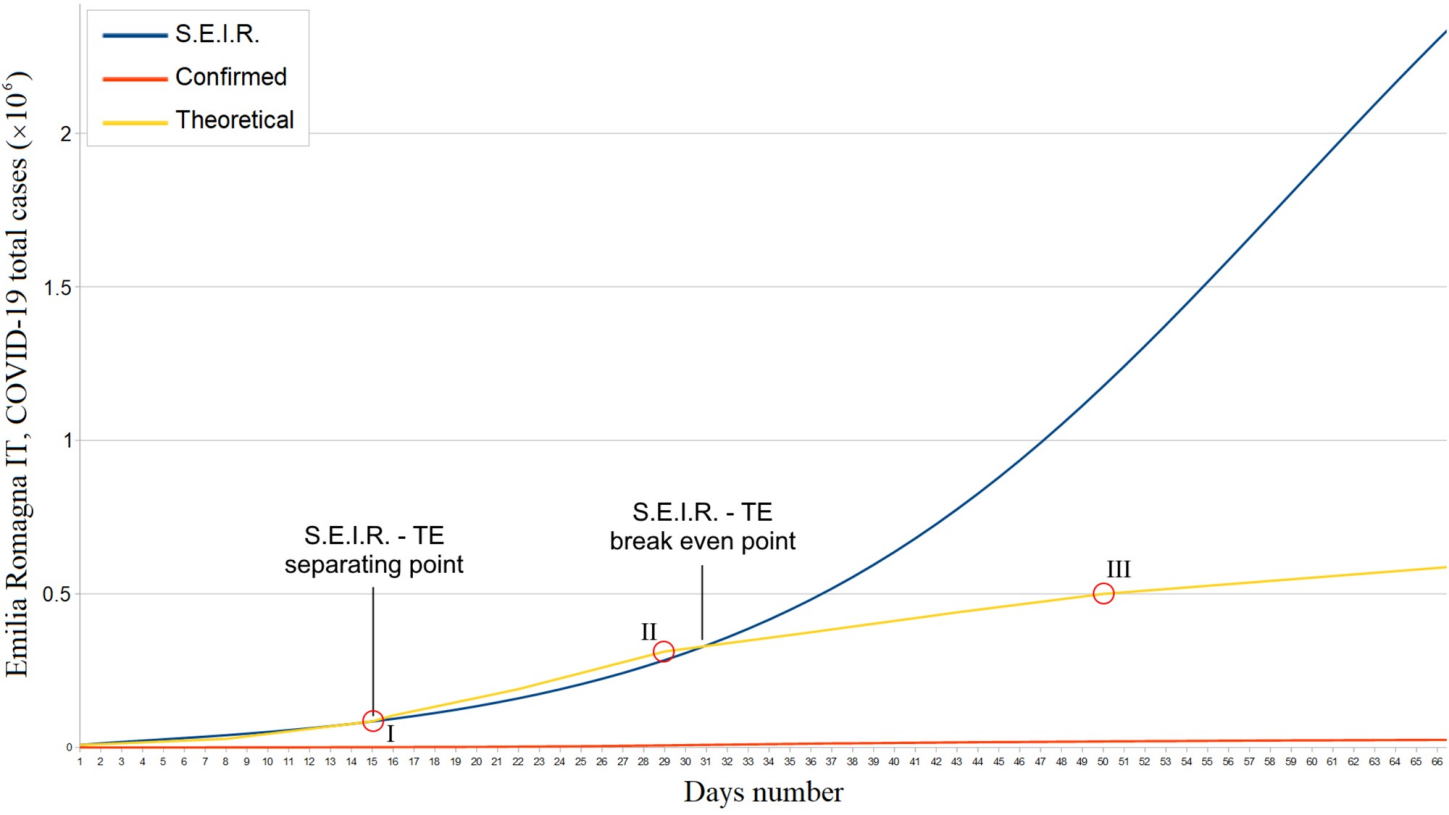
Emilia Romagna confirmed, theoretical, and S.E.I.R. simulation total COVID-19 cases trends from February 29 to April 30, 2020 S.E.I.R. = Susceptible - Exposed - Infectious - Recovered; TE = Theoretical Estimate

About Emilia Romagna, we estimated a basic reproduction number $R0 = 2.22\;(95\% CI: 2.18 - 2.26, CRI: 2.20 - 2.23)$, with $\Delta = 0.09\;(95\% CI: 0.07 - 0.13)$ and $\varepsilon = 0.10\;(95\% CI: 0.08 - 0.12)$ in the same Lombardy investigation period. On May 1, 2020, the estimated number of Emilia Romagna real infections exceeds that of confirmed infections by a factor $\alpha \sim 24\; (\alpha = 23.7, 95\% CI: 23.3 - 23.9)$. The corner point I in Figure 2 signals an $R0$ increase, which lasted approximately until March 21 (Figure 2).

**Figure 3:**
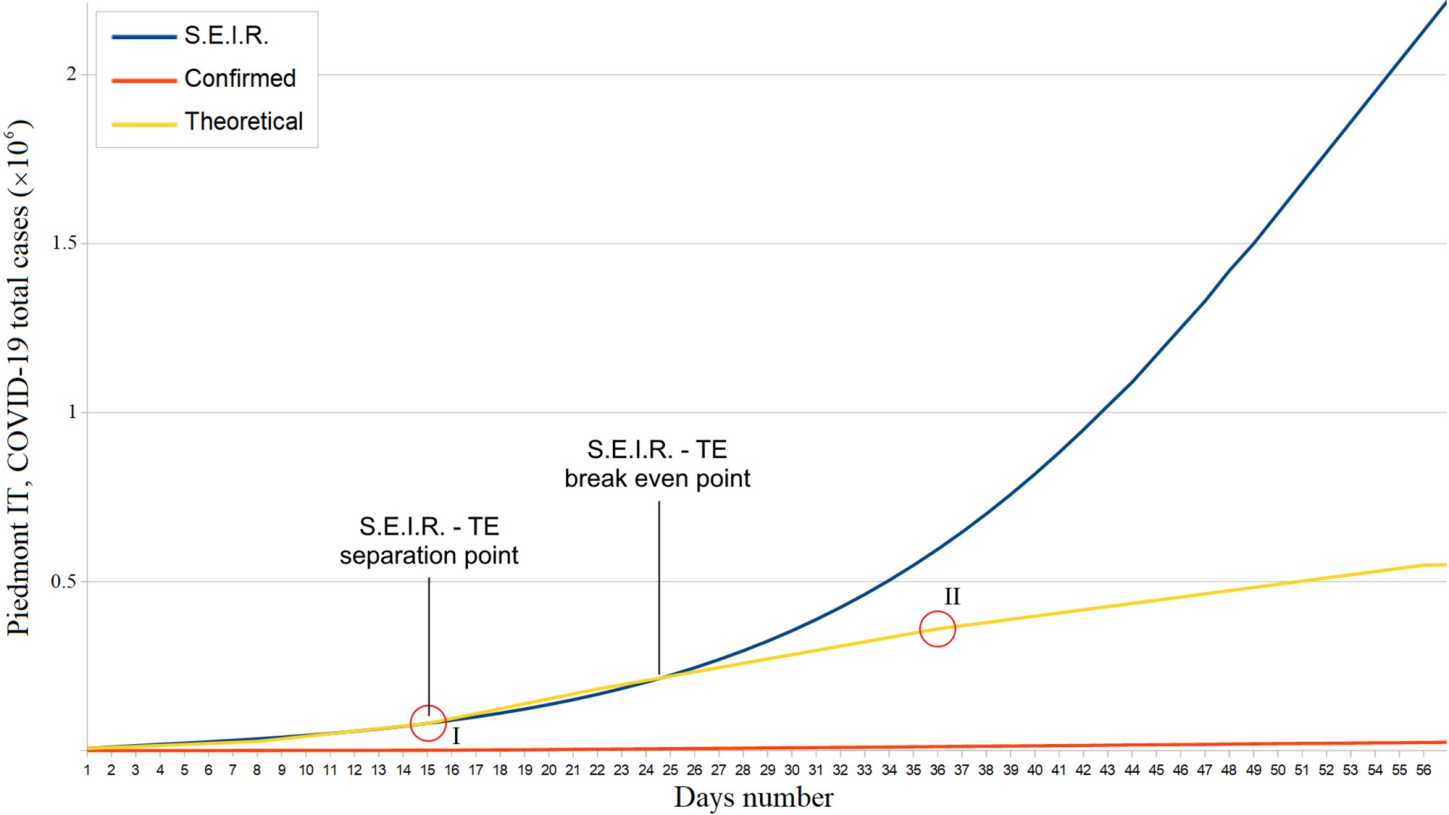
Piedmont confirmed, theoretical, and S.E.I.R. simulation total COVID-19 cases trends from February 29 to April 25, 2020 S.E.I.R. = Susceptible - Exposed - Infectious - Recovered; TE = Theoretical Estimate

About Piedmont, we estimated a $R0 = 2.52\;(95\% CI: 2.48 - 2.56)$, with $\Delta = 0.07\;(95\% CI: 0.01 - 0.13)$ and $\varepsilon = 0.07\;(95\% CI: 0.06 - 0.09)$ from February 29 to March 14, 2020 (Ministry of Health data show the infection appears to have started about a week late in this region). Moreover, an important slope increase has occurred on March 14 (15^th^ day) (Figure 3); this remained almost constant until April 4 (corner point II). On April 25, the estimated COVID-19 total cases in Piedmont exceed those confirmed by a factor $\alpha = 23\;(\alpha = 22.5, 95\% CI: 22.0 - 23.0)$. Between March 23 and 24, we notice the first real positive effects of the lockdown.

Pollution correlation

No significant correlation between SARS-CoV-2 and PM 10 was found (Tables 1-2).

**Table 1:** Top Italian “PM 10 polluted and COVID-19" affected cities PM 10 = Particulate Matter 10; AV = Average Value; CI = Confidence Interval; SEM = Standard Error of the Mean

Region	City	Pre-lockdown (Jan 1, Feb 29)		Lockdown (March 1, May 14)		Population density (P/km²)	Population (x 10^6^)
		PM 10 AV (µg/m³)	95% CI (SEM)	PM 10 AV (µg/m³)	95% CI (SEM)		
Lombardy	Milan	56	50 - 62	26	23 - 29	2063	3.209
	Brescia	50	46 - 55	27	24 - 30	264.5	1.264
	Bergamo	43	38 - 47	21	19 - 24	417.9	1.108
Emilia Romagna	Reggio Emilia	54	49 - 59	26	22 - 29	232.2	0.533
	Piacenza	52	47 - 57	24	21 - 27	111.1	0.287
	Bologna	46	40 - 51	21	18 - 24	2773	1.006
Piedmont	Turin	59	51 - 67	20	18 - 22	331	2.282
	Alessandria	60	54 - 66	26	23 - 30	118.4	0.429
Lazio	Roma	40	38 - 41	23	22 - 24	810	4.354
	Roma Tiburtina	51	48 - 54	21	19 - 22	3600	0.17
	Frosinone	51	48 - 55	22	21 - 23	153.3	0.496
Campania	Napoli	50	48 - 51	27	26 - 27	2617	3.117

**Table 2:** May 14, 2020: top Italian “PM 10 polluted and COVID-19" affected cities correlation matrix PM 10 = Particulate Matter 10

Correlation matrix (p-values)			
1 (0)			
.20 (.56)	1 (0)		
-.22 (.52)	.07 (.84)	1 (0)	
-.30 (.37)	.34 (.31)	.46 (.15)	1 (0)

**Figure 4:**
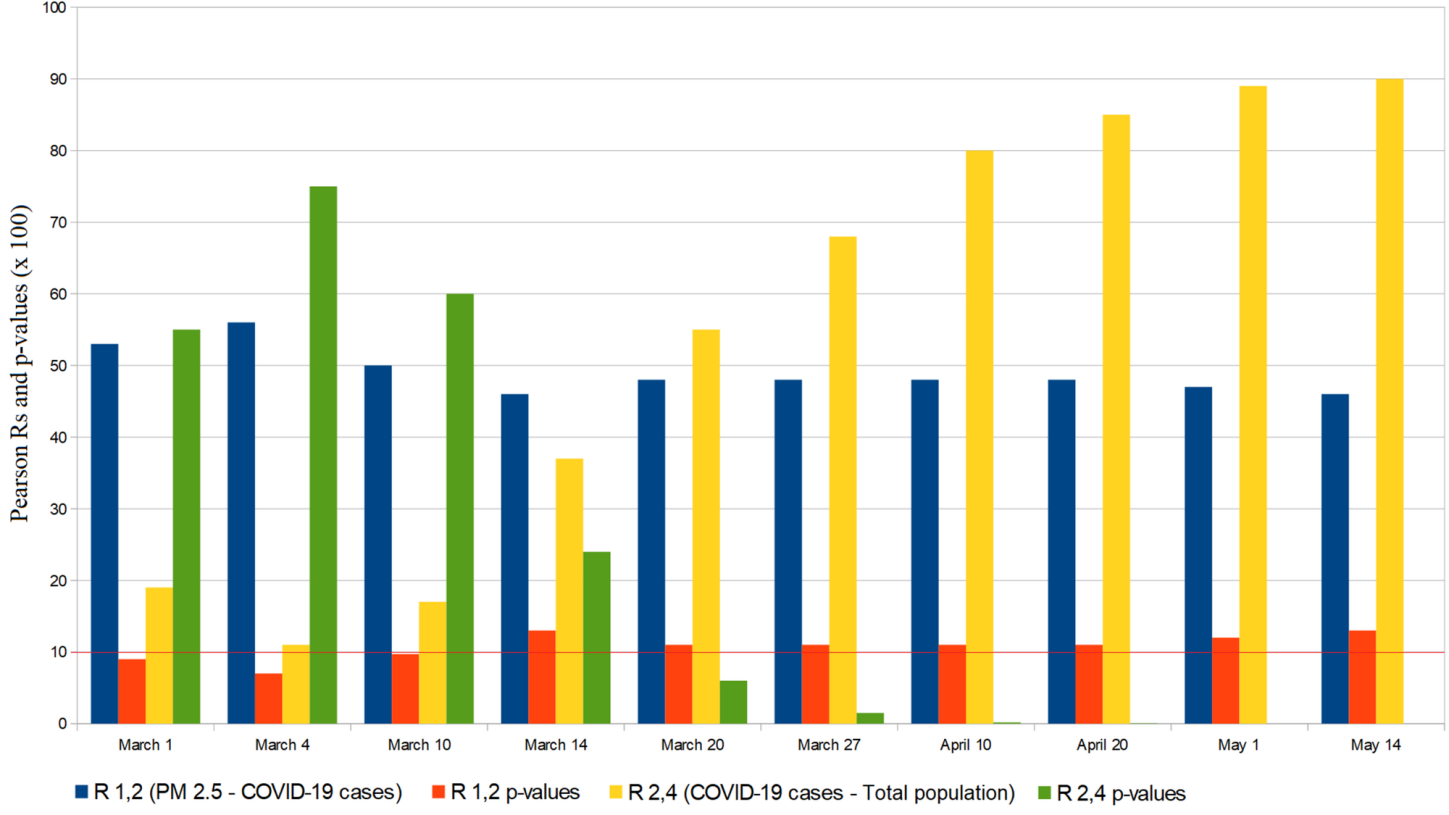
Lombardy cities “PM 2.5 aerosols and populations” correlations with COVID-19 cases R = Pearson correlation value; PM 2.5 = Particulate Matter 2.5

A statistically significant correlation between SARS-CoV-2 spread and PM 2.5 was highlighted in Lombardy during the first two weeks of March, with a $\text{p-value} = .07$ and a Pearson correlation coefficient $\rho = .56$ (Tables 3-4). Beyond that time, the above correlation has decreased in favor of a very significant SARS-CoV-2 - provinces population number correlation ($\text{p-value} &lt; .0001, \rho = 0.9$) (Figure 4). Anyway, as can be seen in Figure 4, we must point out that, excluding the period from March 10-14, the PM 2.5 correlation p-values always remained close to the chosen threshold until the end of April.

**Table 3:** Lombardy cities populations, PM 2.5 daily average values from January 1 to May 14, 2020, and COVID-19 total cases until May 14, 2020 PM 2.5 = Particulate Matter 2.5; AV = Average Value; CI = Confidence Interval; SEM = Standard Error of the Mean

Cities	PM 2.5 (μg/m³)	95% CI (SEM)	COVID-19 cases (May 14)	Population density (P/km²)	Total population (x 10^6^)
Milan	43	39 - 46	22151	2063	3.209
Brescia	41	39 - 44	14147	292.6	1.264
Bergamo	42	39 - 45	12443	264.5	1.108
Cremona	47	44 - 51	6323	204.5	0.362
Monza B.	44	41 - 47	5287	417.9	0.872
Pavia	45	41 - 49	4979	742	0.89
Como	39	36 - 42	3629	418.4	0.339
Varese	32	30 - 35	3379	2228	0.6
Lodi	39	37 - 41	3351	292.6	0.182
Mantova	39	37 - 41	3291	3729	0.872
Lecco	24	21 - 26	2645	177.3	0.415
Sondrio	21	19 - 22	1367	184.7	0.548

**Table 4:** Lombardy cities populations, PM 2.5 daily average values, and COVID-19 total cases: most significant correlation values and days PM 2.5 = Particulate Matter 2.5

Date	Correlation matrix (p-values)			
March 4, 2020	1 (0)			
	.56 (.07)	1 (0)		
	-.08 (.82)	-.33 (.32)	1 (0)	
	-.32 (.34)	.11 (.75)	.32 (.34)	1 (0)
May 14, 2020	1 (0)			
	.46 (.13)	1 (0)		
	.07 (.83)	.09 (.78)	1 (0)	
	.30 (.34)	.90 (.0001)	.35 (.26)	1 (0)

The SARS-CoV-2 - provinces population number correlation in Emilia Romagna grew much more slowly than in Lombardy, reaching its maximum value on May 14, 2020 ($\rho = .57, \text{p-value} = .11$). Finally, in Piedmont, there were significant correlations between the COVID-19 cases and the population number (ρ = .99, p-value <.0001) and, unlike Emilia Romagna and Lombardy, the COVID-19 cases and the population density (ρ = .71, p-value = .048 on May 14, 2020) (Appendix 2).

## Discussion

For this analysis, we made some assumptions:

· given the antibody response identified in COVID-19 patients, it seems unlikely that the novel coronavirus, without significantly changing, could infect a patient again (in the short term) [11-12];

· the mortality rate is too low to affect the evolution of the S.E.I.R. system [7-9];

· Young people and children appear to have an important role in the spread of infection [13-15].

Therefore, to analyze the SARS-CoV-2 dynamics in the initial stages, we have adopted the S.E.I.R. model (Susceptible → Exposed → Infectious → Recovered) since it is suitable for describing the spread of a virus in a non-relapse free population. This allowed us to evaluate the effectiveness of the containment measures and/or any virus behavior mutation by comparing S.E.I.R. and theoretical trends (Figures 1-2). Given the absolutely abnormal number of COVID-19 infections and deaths, we treated Lombardy as a standalone case; moreover, the modeling we have made exclusively concerns northern Italy since it was the most COVID-19 affected region. Our results show the effectiveness of the Italian lockdown. However, the strong correlation between the total infected and the number of inhabitants in Lombardy suggests the virus nevertheless circulated in a very substantial way among this region, i.e., containment measures are likely to have been taken with a heavy delay. In fact, as of May 1, the estimated number of total COVID-19 cases in Lombardy was almost 3 million. Furthermore, it is plausible that the effect of such a latency was aggravated by the presence of PM 2.5 as shown in Figure 4. In this regard, we must point out the COVID-19 cases - PM 2.5 correlation p-value has never exceeded a maximum of .16. The substantial difference between the basic reproduction number of Lombardy and that of the other two most affected regions is the main symptom of the local behavior of the novel coronavirus. About this aspect, various speculations, hypotheses, and theories were made: some of them concern a possible evolutionary genetic mutation of SARS-CoV-2, which has made it more contagious or virulent [16-19]. If so, this should have afflicted Lombardy. Other researches instead assert that the virus's genetic mutations did not cause a mutation in its behavior [20]; if so, the greater Lombard virulence should find explanations in correlation with pollution, delay in lockdown, or other factors such as a major predisposition of the subjects to be infected and develop severe symptoms [10,21]. However, it must be considered that many of these scenarios could prove to be true simultaneously. One hypothesis to be excluded is the bijective relation between local demography and SARS-CoV-2 spread: in fact, Lombardy and Emilia Romagna's age groups are totally comparable [22-23]. The behavior of the novel coronavirus in Piedmont was also peculiar: first, despite what happened in Lombardy and Emilia Romagna, we found a strong correlation between the population density and the number of total cases. Furthermore, the epidemic seems to have started with a delay of one week. This fact cannot be explained by the presence of particulate pollution since the concentrations of PM 2.5 and PM 10 were already below the safety threshold due to the quarantine. Therefore, this episode could be linked to virus behavior mutations, inadequate containment measures, or people lockdown violations. As for a possible link between PM 10 and novel coronavirus, we found no significant correlation. At the national level, concentrations of PM 10 in some cities of central and southern Italy were comparable to those of Lombardy and Emilia Romagna. Cities like Frosinone, Rome Tiburtina, and Naples, where COVID-19 infections were few, had higher values than cities like Brescia and Bergamo where the infection was devastating. Moreover, PM 10 concentrations in Emilia Romagna and Piedmont are equivalent to Lombard ones unlike the density of COVID-19 cases. Evaluating the hypothesis that the first outbreak location was highly incident on the virus spread, we conducted the analysis only in the Lombardy region, but the result was still negative. The lack of a clear correlation, however, is not sufficient to exclude every relation between PM 10 and SARS-CoV-2 for two reasons:

· the town of Codogno, where the first Lombardy outbreak occurred, recorded very high PM 10 average daily values between January 1 and February 29 ($67 \mu g / m^3$) (Appendix 2);

· the novel coronavirus behavior may not be related to the daily average values of PM 10 but to specific thresholds, above which its virulence and contagiousness would increase considerably. For example, an hour of very heavy traffic could favor its spread much more than a constant release of the same particulate matter amount over a longer time-lapse.

On the contrary, both at the national level (as shown by Frontera et al.) and in Lombardy, the correlation with PM 2.5 was much more evident and significant. For this reason, further investigations are required to determine what is the biological link between the spread of SARS-CoV-2 and fine pollution.

Limitations

The S.E.I.R. model predictability is inversely proportional to the prediction temporal distance since the fixed values, such as basic reproduction number, incubation time, and healing time, could change due to virus behavior mutations; furthermore, iterative methods propagate errors divergently. Anyway, its adoption in the short term remains valid since error propagation is truncated. The mortality rate is considered constant in the period February 22 - May 1, 2020. Regarding PM 10 and PM 2.5 aerosols, we only had access to cities' and regions' daily average values and the data of some monitoring stations were not available. One study used to estimate real COVID-19 cases had no peer review; however, the data inherent to the virus mortality were also supported by peer-reviewed articles and through an independent verification from the National Institute of Statistics (ISTAT) website [24].

## Conclusions

In this study, we demonstrated that the intra-regional and provincial analysis is able to highlight substantial differences in the behavior of the novel coronaviruses. In particular, in Lombardy, we found a significant correlation between virus spread and the number of inhabitants per province while in Piedmont, this happened also with the population density. Significant correlations occurred in Lombardy between the number of COVID-19 cases per province and the presence of PM 2.5; this confirms what has been observed in other studies on a national scale. Our analysis of the real number of infected leads us to conclude that the Civil Protection official data are enormously underestimated. From the comparison between the S.E.I.R predictions and the calculated real infections trends, as well as between the infections trends of Lombardy and other Italian regions, we report that the Italian COVID-19 data are statistically compatible with possible evolutionary mutations of SARS-CoV-2. Although a statistical investigation is absolutely essential to understand the behavior of SARS-CoV-2, it cannot be a substitute for a molecular investigation. For this reason, we believe this paper can be useful to guide biologists and virologists on the virus specificities that need to be further explored, thus preventing the return of such a crisis.

## Appendices

Appendix 1

#include <iostream>

#include <math.h>

#include <cmath>

using namespace std;

int main ()

{

double I1,I2,N,S,E1,E2,R,sigma,gamma,R0,beta,delta,t=0,T;

//const double Ndays=30; // set it if you want to print values until a specific day

int i=1;

const double min_I = 0.5; // minimum Infected-number we want to achieve

I2 = ; // setting initial infections

R = ; // setting initial recoveries

N = ; // setting population

sigma = 3.85802e-6; // incubation rate

gamma = 8.2672e-7; // recovery rate

R0 = ; // setting basic reproduction number

E2 = R0*I2; // theoretical estimate of incubated people

S = N - E2 - I2 - R;

beta = R0*gamma;

delta = 0.05; // max value recommended = 1

//T = Ndays*86400; // use it if you want to print values until a specific day

do

{

    E1 = E2;

    I1 = I2;

    E2 = (beta*I1*S/N-sigma*E1)*delta+E1;

    I2 = (sigma*E1-gamma*I1)*delta+I1;

    R = gamma*I1*delta+R;

    S = -beta*I1/N*S*delta+S;

    t = t + delta;

    if (t>=86400*i)

    {

        i++;

        //cout << " day " << i << endl << endl;

        /*cout << " N = " << N << endl << endl;

        cout << " S = " << S << endl << endl;

        cout << " E2 = " << E2 << endl << endl;*/

        //cout << " I2 = " << endl << endl;

        //cout << " R = " << R << endl << endl;

        cout << I2+R<< endl;

    }

} while (I2>min_I); // I print values until it drops to a minimum daily infected value

//while (t<=T); // I print values until a specific day

cout << "v2.04" << endl;

//cout << "Last day total cases = " << I+E+R << endl;

int x;

cin >> x; //terminate program

return 0;

}

Appendix 2

**Table 5:** Lombardy cities PM 10 values from Jan 1, to Feb 29, 2020, COVID-19 cases on May 14, 2020, correlations PM: particulate matter

Cities	PM 10 (μg/m³)	95% CI (SEM)	COVID-19 cases	Population density (P/km²)	Total population (x 10^6)	ρ PEARSON 1.2
Milan	56	50 - 62	22151	2063	3.209	0.29
Brescia	50	46 - 55	14147	292.6	1.264	ρ PEARSON 2.3
Bergamo	43	38 - 47	12443	264.5	1.108	0.09
Cremona	61	56 - 67	6323	204.5	0.362	ρ PEARSON 1.3
Monza e Brianza	57	51 - 63	5287	417.9	0.872	-0.18
Pavia	55	50 - 61	4979	742	0.89	ρ PEARSON 1.4
Como	48	43 - 53	3629	418.4	0.339	0.15
Varese	46	41 - 51	3379	2228	0.6	ρ PEARSON 2.4
Lodi (Codogno 67)	62	56 - 70	3351	292.6	0.182	0.90
Mantova	39	37 - 41	3291	3729	0.872	ρ PEARSON 3.4
Lecco	34	30 - 39	2645	177.3	0.415	0.35
Sondrio	36	33 - 40	1367	184.7	0.548	May 14

**Table 6:** Lombardy cities PM 10 values from Jan 1 to Feb 29, 2020, COVID-19 cases on March 4, 2020, correlations PM: particulate matter

Cities	PM 10 (μg/m³)	95% CI (SEM)	COVID-19 cases	Population density (P/km²)	Total population (x 10^6)	ρ PEARSON 1.2
Milan	56	50 - 62	145	2063	3.209	0.36
Brescia	50	46 - 55	127	292.6	1.264	ρ PEARSON 2.3
Bergamo	43	38 - 47	423	264.5	1.108	-0.33
Cremona	61	56 - 67	333	204.5	0.362	ρ PEARSON 1.3
Monza e Brianza	57	51 - 63	11	417.9	0.872	-0.12
Pavia	55	50 - 61	126	742	0.89	ρ PEARSON 1.4
Como	48	43 - 53	154	418.4	0.339	0.31
Varese	46	41 - 51	11	2228	0.6	ρ PEARSON 2.4
Lodi (Codogno 67)	excluded as overestimating due to the high number of swabs made					0.11
Mantova	39	37 - 41	22	3729	0.872	ρ PEARSON 3.4
Lecco	34	30 - 39	5	177.3	0.415	0.32
Sondrio	36	33 - 40	4	184.7	0.548	May 14

**Table 7:** Emilia Romagna provinces COVID-19 cases correlation with population density and population number

Cities	COVID-19 total cases	Pop. Density [1]	Population [2]	ρ 1	ρ 2
Bologna	2	2773	1.01E+006	-0.2031158263	-0.3428304313
Ferrara	0	131.6	351436	p-value 1	p-value 2
Forlì-Cesena	1	165.9	398322	.61	.37
Modena	24	262.4	701642	March 1	2020
Parma	59	758.29	447779		
Piacenza	174	113	286997		
Ravenna	2	211	389880		
Reggio nell'Emilia	7	363	532872		
Rimini	16	386	335463		
Cities	COVID-19 total cases	Pop. Density [1]	Population [2]	Pearson 1	Pearson 2
Bologna	11	2773	1.01E+006	-0.1800698791	-0.3299337657
Ferrara	0	131.6	351436	p-value 1	p-value 2
Forlì-Cesena	2	165.9	398322	.64	.39
Modena	41	262.4	701642	March 4	2020
Parma	115	758.29	447779		
Piacenza	319	113	286997		
Ravenna	2	211	389880		
Reggio nell'Emilia	20	363	532872		
Rimini	33	386	335463		
	1				
Cities	COVID-19 total cases	Pop. Density [1]	Population [2]	Pearson 1	Pearson 2
Bologna	86	2773	1.01E+006	-0.1196856068	-0.3011957866
Ferrara	8	131.6	351436	p-value 1	p-value 2
Forlì-Cesena	20	165.9	398322	.78	.43
Modena	127	262.4	701642	March 10	2020
Parma	325	758.29	447779		
Piacenza	633	113	286997		
Ravenna	24	211	389880		
Reggio nell'Emilia	104	363	532872		
Rimini	206	386	335463		
Cities	COVID-19 total cases	Pop. Density [1]	Population [2]	Pearson 1	Pearson 2
Bologna	2954	2773	1.01E+006	0.3384088596	0.4767428453
Ferrara	566	131.6	351436	p-value 1	p-value 2
Forlì-Cesena	1099	165.9	398322	.37	.19
Modena	2930	262.4	701642	April 10	2020
Parma	2473	758.29	447779		
Piacenza	3049	113	286997		
Ravenna	776	211	389880		
Reggio nell'Emilia	3630	363	532872		
Rimini	1651	386	335463		
Cities	COVID-19 total cases	Pop. Density [1]	Population [2]	Pearson 1	Pearson 2
Bologna	3822	2773	1.01E+006	0.4224336429	0.5599738639
Ferrara	787	131.6	351436	p-value 1	p-value 2
Forlì-Cesena	1435	165.9	398322	.26	.11
Modena	3411	262.4	701642	April 20	2020
Parma	2887	758.29	447779		
Piacenza	3393	113	286997		
Ravenna	934	211	389880		
Reggio nell'Emilia	4352	363	532872		
Rimini	1846	386	335463		
Cities	COVID-19 total cases	Pop. Density [1]	Population [2]	Pearson 1	Pearson 2
Bologna	4458	2773	1.01E+006	0.4492972218	0.5532168163
Ferrara	926	131.6	351436	p-value 1	p-value 2
Forlì-Cesena	1586	165.9	398322	.22	.12
Modena	3678	262.4	701642	May 1	2020
Parma	3177	758.29	447779		
Piacenza	4115	113	286997		
Ravenna	982	211	389880		
Reggio nell'Emilia	4713	363	532872		
Rimini	2009	386	335463		
Cities	COVID-19 total cases	Pop. Density [1]	Population [2]	Pearson 1	Pearson 2
Bologna	4841	2773	1.01E+006	0.4748711825	0.5665261751
Ferrara	977	131.6	351436	p-value 1	p-value 2
Forlì-Cesena	1695	165.9	398322	.19	.11
Modena	3839	262.4	701642	May 14	2020
Parma	3346	758.29	447779		
Piacenza	4405	113	286997		
Ravenna	1000	211	389880		
Reggio nell'Emilia	4870	363	532872		
Rimini	2083	386	335463		

**Table 8:** Piedmont provinces COVID-19 cases correlation with population density and population number

COVID-19 cases	Pop. Density [I]	Population [II]	Pearson I
0	118.4	428826	0.706
0	142.1	217574	p-value I
0	192.3	179685	.048
0	85.2	590421	Pearson II
0	275.3	370525	-0.21
49	331	2282000	p-value II
0	70	157782	.62
0	82.1	174904	
COVID-19 cases	Pop. Density [I]	Population [II]	Pearson I
16	118.4	428826	-0.002
41	142.1	217574	p-value I
0	192.3	179685	1
0	85.2	590421	Pearson II
3	275.3	370525	0.00
11	331	2282000	p-value II
5	70	157782	1
3	82.1	174904	
COVID-19 cases	Pop. Density [I]	Population [II]	Pearson I
65	118.4	428826	0.600
58	142.1	217574	p-value I
20	192.3	179685	.11
14	85.2	590421	Pearson II
22	275.3	370525	0.81
111	331	2282000	p-value II
11	70	157782	.015
18	82.1	174904	
COVID-19 cases	Pop. Density [I]	Population [II]	Pearson I
2119	118.4	428826	0.693
703	142.1	217574	p-value I
628	192.3	179685	.06
1318	85.2	590421	Pearson II
1258	275.3	370525	0.99
7226	331	2282000	p-value II
790	70	157782	<.00001
708	82.1	174904	
COVID-19 cases	Pop. Density [I]	Population [II]	Pearson I
2815	118.4	428826	0.707
1142	142.1	217574	p-value I
787	192.3	179685	.048
2075	85.2	590421	Pearson II
1996	275.3	370525	0.99
10278	331	2282000	p-value II
937	70	157782	<.00001
984	82.1	174904	
COVID-19 cases	Pop. Density [I]	Population [II]	Pearson I
3491	118.4	428826	0.708
1600	142.1	217574	p-value I
970	192.3	179685	.048
2493	85.2	590421	Pearson II
2333	275.3	370525	0.99
13311	331	2282000	p-value II
1022	70	157782	<.00001
1117	82.1	174904	
COVID-19 cases	Pop. Density [I]	Population [II]	Pearson I
3769	118.4	428826	0.709
1711	142.1	217574	p-value I
1022	192.3	179685	.048
2666	85.2	590421	Pearson II
2543	275.3	370525	0.99
14807	331	2282000	p-value II
1099	70	157782	<.00001
1226	82.1	174904	
