# Correction: Modeling the Epidemiological Trend and Behavior of COVID-19 in Italy

**DOI:** 10.7759/cureus.c37

**Published:** 2020-09-11

**Authors:** Alessandro Rovetta, Akshaya S Bhagavathula, Lucia Castaldo

**Affiliations:** 1 Mathematical, Statistical and Epidemiological Models, Research and Disclosure Division, Mensana Srls, Brescia, ITA; 2 Mathematical, Statistical and Epidemiological Models, Technological and Scientific Research, Redeev Srl, Naples, ITA; 3 Public Health, Institute of Public Health, College of Medicine and Health Sciences, United Arab Emirates University, Al Ain, ARE; 4 Mathematics, Technological and Scientific Research, Redeev Srl, Naples, ITA; 5 Mathematics, Research and Disclosure Division, Mensana Srls, Brescia, ITA

Due to a malfunction of the data reading of the software used for the calculations, the authors have reported a wrong datum in the publication. The error alters the results of the study in an absolutely limited way: in fact, it is only one of the dozens of calculated correlations; all other correlations have been re-checked and are correct. Furthermore, this result does not in any way affect the estimate of the R0s of the various regions or the estimate of the real number of infected (appropriately re-checked).

The following changes have been made to the article as a result:

1. Results section:
  Original sentence: "Finally, in Piedmont, there was a significant correlation between the COVID-19 cases and the population density (ρ = .71, p-value = .048 on May 14, 2020) unlike Emilia Romagna and Lombardy (Appendix 2 ). No SARS-CoV-2 - province population number correlation was found there."
  New sentence: "Finally, in Piedmont, there were significant correlations between the COVID-19 cases and the population number (ρ = .99, p-value <.0001) and, unlike Emilia Romagna and Lombardy, the COVID-19 cases and the population density (ρ = .71, p-value = .048 on May 14, 2020) (Appendix 2).
2. Conclusions section:
  Original sentence: "In particular, in Lombardy, we found an extremely significant correlation between virus spread and the number of inhabitants per province while in Piedmont, this happened only with the population density."
  New sentence: "In particular, in Lombardy, we found a significant correlation between virus spread and the number of inhabitants per province while in Piedmont, this happened also with the population density."
3. Table 8 (in Appendix 2)​​​​​​​ Format fixes: - all commas (indicating the beginning of the decimal part of a number in Italian notation) have been replaced with periods (indicating the beginning of the decimal part of a number in English notation) - all the reported values now contain, when necessary, a maximum of 3 significant digits in the decimal part Content corrections: - the value "Pearson II = -0.34" of the second sub-table has been replaced with the value "Pearson II = 0.00" - the value "p-value II = .41" of the second sub-table has been replaced with the value "p-value II = 1" - the value "Pearson II = -0.42" of the third sub-table has been replaced with the value "Pearson II = 0.81" - the value "p-value II = .30" of the third sub-table has been replaced with the value "p-value II = .015" - the value "Pearson II = -0.31" of the fourth sub-table has been replaced with the value "Pearson II = 0.99" - the value "p-value II = .46" of the fourth sub-table has been replaced with the value "p-value II <.0001" - the value "Pearson II = -0.34" of the fifth, sixth, and seventh sub-table has been replaced with the value "Pearson II = 0.99" - the value "p-value II = .41" of the fifth, sixth, and seventh sub-table has been replaced with the value "p-value II <.0001"

 

